# Association between social capital and frailty and the mediating effect of health-promoting lifestyles in Chinese older adults: a cross-sectional study

**DOI:** 10.1186/s12877-022-02815-z

**Published:** 2022-03-02

**Authors:** Shan Hu, Canhuan Jin, Shaojie Li

**Affiliations:** 1 Hunan Cancer Hospital, The Affiliated Cancer Hospital of Xiangya School of Medicine, Central South University, Changsha, 410013 China; 2 Department of Social Medicine and Health Service Management, Xiangya School of Public Health, Central South University, Changsha, 410078 China

**Keywords:** Social capital, Health-promoting lifestyles, Frailty, Older adults

## Abstract

**Background:**

To explore the association between social capital and frailty and the mediating effect of health-promoting lifestyles among Chinese older adults, while providing scientific evidence for frailty intervention.

**Methods:**

In May 2021, a cross-sectional study was conducted among 674 Chinese older adults in Changsha city. Data was collected using the Chinese Shortened Social Capital Scale (comprising structural social capital and cognitive social capital as two subscales), a simplified version of the Health-Promoting Lifestyle Profile and the Tilburg Frailty Indicator. Linear regression analysis was used to examine the association between social capital and frailty. Structural equation modeling was used to test the mediating effect of health-promoting lifestyles.

**Results:**

Cognitive social capital was significantly negatively associated with frailty and its three dimensions (physical, psychological, and social frailty), but structural social capital was not. Health-promoting lifestyles played a mediating role in the associations of cognitive social capital with frailty, physical and psychological frailty, but not with social frailty.

**Conclusions:**

Higher cognitive social capital was associated with a reduced likelihood of frailty. The health-promoting lifestyles partially mediated the association between cognitive social capital and frailty. The use of health-promoting lifestyles or appropriate cognitive social capital interventions may reduce frailty among older adults.

**Supplementary Information:**

The online version contains supplementary material available at 10.1186/s12877-022-02815-z.

## Background

The aging of a population is a major social problem worldwide, affecting all aspects of the economy, politics, social development, and the health care system [1]. In the field of public health, the prevention of aging-related diseases (including cognitive impairment, disability, frailty, etc.) is a trending topic. Among them, as a disease syndrome closely related to age, frailty has become an important criterion for the evaluation of the health status of older adults [2]. The core feature of frailty is the decline in the physiological capacity of multiple organ systems, leading to increased susceptibility to stressors [3]. Meta-analysis results have indicated that frailty increases the risk of a series of negative health outcomes [4], such as mortality [5], hospitalization [6], disability [7], falls [8], fractures [9], and dementia [10]. In recent years, with the increase in the aging population, the incidence and prevalence of frailty has also been increasing. The results of a systematic review indicated that among non-frail individuals with a median follow-up time of 3 years, the combined incidence of frailty was 43.4 per thousand person-years [11]. A meta-analysis of older adults in communities in low-income and middle-income countries revealed that the combined prevalence of frailty was 17.4%, and the prevalence of prefrailty was 49.3% [12]. Considering the negative health status caused by the high incidence and prevalence of frailty and related factors, should be explored to provide scientific evidence for the prevention of, and intervention for, frailty in older adults.

The mechanism of the occurrence and development of frailty is more complicated, and is the result of the interaction of many factors. At present, a considerable number of studies have explored the factors related to frailty in older adults. A literature review revealed that sociodemographic factors (such as sex, age, education level, and income) [13], physiological factors (such as genes, inflammatory factors, body composition, and malnutrition) [14–17], chronic diseases (such as diabetes, cardiovascular disease, and stroke) [18], lifestyles (such as physical activity, sedentary lifestyle, fruit and vegetable consumption, and dietary patterns) [19–21] and psychological factors (such as stress, depression, and anxiety) [22–24] may cause frailty. In addition, studies have also found that social factors such as social isolation [25], frequency of interaction with relatives and friends [26], and social support [27] were associated with frailty. However, the social factors in these studies were predominantly examined at individual level, and few studies investigated the association between overall social factors, such as social capital, and frailty [28, 29].

Social capital refers to the social resources and benefits that individuals obtained through contact with others [30], and are usually divided into two aspects: Structural social capital and cognitive social capital [31]. The structural aspect refers to externally observable social resources, such as social networks and social participation, and the cognitive aspect reflects the subjective attitude and evaluation of social relations, such as trust and the norms of reciprocity [31]. Social capital is considered to be a positive social determinant of health [32], and a large number of studies have confirmed that it is significantly related to physical and mental health [33, 34]. In addition, increased evidence indicates that interventions related to social capital can significantly improve the health of older adults and promote healthy aging [35]. A previous review emphasized the need to investigate the impact of social capital on frailty in older adults in the context of the rapid growth in global life expectancy and aging [36]. However, there is limited empirical research exploring the relationship between social capital and frailty in older adults. Previous studies on the frailty and aging cohort in Korea have indicated that insufficient social capital is significantly related to frailty [28]. However, the study defined insufficient social capital as a lack of participation in social gatherings [28], and Japanese research on frailty factors only reflected social capital from three aspects: social participation in activities, trust in the community, and interaction with neighbors [29]. These definitions and assessment contents were evidently one-sided and failed to reflect the true meaning of social capital. Therefore, it is necessary to adopt more comprehensive social capital assessment tools to better explore its association with frailty.

In addition, to better prevent and intervene with regard to frailty in older adults from the social capital perspective, the potential mediating factors in the relationship between to social capital and frailty should be understood. The model of the social determinants of health maintains that personal health is primarily affected by four aspects, namely, 1) general social factors, such as social economy, culture, and environment, 2) living and working conditions, 3) community network, and 4) personal lifestyles [37]. Among them, social factors are considered to be upstream determinants, that can have an indirect impact on individual health through downstream lifestyle factors [38]. Previous studies have found that social capital can indirectly affect health through lifestyles [39]. However, it remains unknown whether the mediating role of healthy lifestyles can be generalized to the relationship between social capital and frailty. Considering that lifestyles are significantly related to social capital and frailty [40, 41], the above-mentioned mediation effect may also be established, but there is no research to confirm it.

Therefore, the objective of this study was to investigate the association between social capital (including structural and cognitive social capital) and frailty (including physical, psychological, and social frailty) of older adults. We also explored the potential mediating role of health-promoting lifestyles in the association between social capital and frailty. We developed the following priori hypotheses: 1) Social capital will be positively correlated with frailty; 2) Health-promoting lifestyles will mediate the relationship between social capital and frailty.

## Methods

### Participants

The formula for an epidemiologic study for estimating population rate $\Big(n=\frac{{Z_{\alpha /2}}^2\pi \left(1-\pi \right)}{\delta^2}$) was used to calculate sample size [42]. According to a previous systematic review, the prevalence of frailty in the older adult population in China was 12.8% [43]. Thus, we set π as 0.128 in this study. If α was 0.05, Z_α/2_ was 1.96, and δ was 0.15, the calculated sample size was 476. To prevent an invalid survey sample, we increased the sample by 20%, which made the minimum sample size in the survey 571. The stratified cluster sampling method was used for sample selection. In May 2021, three districts or counties were randomly selected from six districts and three counties in Changsha City. Consequently, we randomly selected two communities from the three districts or counties, respectively, and surveyed all the older adults in these six communities. The inclusion criteria for the participants were: age greater than or equal to 60 years; local household registration; voluntary participation in the survey. The exclusion criteria were: suffering from serious physical and psychological diseases (reported by family members, such as schizophrenia, dementia, and depression); hearing and vision impairments. University students who had undergone uniform survey training conducted face-to-face interviews with the participants.

### Measures

The questionnaire contained questions regarding demographic characteristics, social capital, health-promoting lifestyles, and frailty. No one of the questionnaires or scales used in our study require a license in order to administer them.

#### Chinese shortened social capital scale (CSSCS)

The CSSCS, which is widely used to assess the social capital of Chinese older adults [40, 44, 45], is a 22-item 5-point Likert (ranging from 1 = “very inconsistent” to 5 = “very consistent) scoring scale. The scale includes two subscales of structural social capital (11 items, comprising three dimensions: social participation, social support, and social connection) and cognitive social capital (11 items, comprising three dimensions: trust, cohesion, and reciprocity). The sum of all items is the total score. The higher the score, the greater social capital of the participant. The overall Cronbach’s α coefficient of CSSCS is 0.92, and it has high construct validity [45].

#### Simplified version of the health-promoting lifestyle profile (HPLP-S)

The HPLP-S is a 24-item scale comprising six dimensions (included four items, respectively): self-actualization, health responsibility, exercise, nutrition, interpersonal support, and stress management. The scale is a simplified version of the 48-item HPLP [46, 47], and each item is scored on a scale of 1 = “never” to 4 = “always.” The total score is calculated by summing the scores of 24 items, and ranges from 24 to 96 points. The higher the total score, the healthier the lifestyle. The Cronbach’s α coefficient of HPLP-S is 0.90, and it demonstrates good construct validity [48].

#### Tilburg frailty Indicator (TFI)

The TFI, which is widely used to assess the frailty in community-dwelling older adults [49], is a 15-item scale comprising three dimensions: physical (eight items), psychological (four items), and social frailty (three items). Each item is scored across two categories [50], that is, “No” is assigned 0 points, and “Yes” is assigned 1 point. The sum of 15 items is the total score of the TFI, and ranges from 0 to 15 points. The higher the score, the more severe the participant’s frailty. A total score greater than or equal to 5 indicates participant frailty. In this study, we used the Chinese version of the TFI, which has been verified among older adults in the community, and has good reliability (Cronbach’s α is 0.71 and test-retest reliability is 0.88) and validity (criterion validity with the Area Under Curves regarding physical phenotype and a frailty index of 0.87 and 0.86) [51].

#### Demographic factors

A self-designed demographic information questionnaire (Additional file 1) was used to collect data on participants’ age (60–69, 70–79, and ≥ 80 years), sex, residence (urban or rural), education level (primary school and below, junior middle school, high school, university and above), marital status (unmarried or married), monthly family income (< 5000, 5000-10,000, > 10,000), and self-rated health (poor, moderate, good).

### Data analysis

Continuous variables conforming to normal distribution (such as social capital, health-promoting lifestyle, and frailty) were described by means ± standard deviation (SD), and categorical variables (such as sex, residence, etc.) were described by N (%). Pearson’s correlation was used to analyze the relationship between social capital, health-promoting lifestyles, and frailty. Linear regression was used to analyze the associations between social capital and frailty and the three dimensions of frailty after adjusting for demographic factors. The above analysis was all conducted using SPSS version 25 (IBM SPSS Statistics, Armonk, NY, USA).

**Fig. 1:**
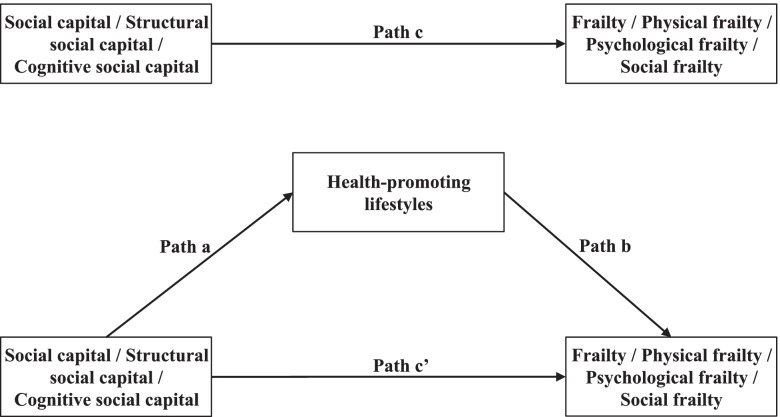
The hypothesised model

The hypothesized mediation model (Fig. 1) test used Amos 24.0 to construct structural equation models (SEM) with bootstrap methods with 95% confidence interval (CI) estimation based on bias correction. The principle of mediation analysis and relevant guidelines indicated that the total, direct and indirect effect were all significant [52, 53]. First, in this study, the path from social capital to frailty (path c, Fig. 1) was significant; second, the path from social capital to health-promoting lifestyles (path a) was significant; third, controlling for social capital, the path from health-promoting lifestyles to frailty (path b) was also significant; and finally, the indirect effect of health-promoting lifestyles (a*b) in the association between social capital and frailty was significant (the 95% CI did not include 0).

## Results

### Descriptive statistics

Descriptive analysis and differences in social capital, health-promoting lifestyles, and frailty scores among participants with different demographic characteristics are illustrated in Table 1.

**Table 1:** Descriptive analysis and differences in social capital, health-promoting lifestyles, and frailty scores among participants with different demographic characteristics (*N* = 674)

Characteristics	***N*** (%)	Mean ± SD				
		Social capital	Structural social capital	Cognitive social capital	Health -promoting lifestyles	Frailty
**Age (years)**						
60–69	347(51.5)	74.66 ± 12.48	32.58 ± 6.61	42.08 ± 7.89	63.59 ± 12.13	2.98 ± 2.02
70–79	270(40.1)	74.87 ± 13.59	32.52 ± 6.98	42.35 ± 8.62	64.77 ± 13.01	3.56 ± 2.53
≥ 80	57(8.5)	72.46 ± 18.72	32.56 ± 10.79	39.89 ± 10.24	63.98 ± 16.71	4.53 ± 2.80
**Sex**						
Male	341(50.6)	74.24 ± 13.25	32.59 ± 7.20	41.65 ± 8.16	63.12 ± 12.69	3.46 ± 2.38
Female	333(49.4)	74.88 ± 13.86	32.52 ± 7.18	42.37 ± 8.66	65.10 ± 13.10	3.23 ± 2.30
**Residence**						
Urban area	285(42.3)	73.84 ± 13.90	32.32 ± 7.38	41.52 ± 8.81	64.48 ± 13.58	4.18 ± 2.70
Rural area	389(57.7)	75.08 ± 13.28	32.72 ± 7.04	42.36 ± 8.11	63.82 ± 12.42	2.73 ± 1.82
**Education level**						
Primary school and below	394(58.5)	73.66 ± 13.66	32.00 ± 6.90	41.66 ± 8.54	62.64 ± 12.18	3.51 ± 2.46
Junior middle school	176(26.1)	74.74 ± 12.36	32.78 ± 6.58	41.97 ± 8.07	63.69 ± 11.65	3.02 ± 1.97
High school	82(12.2)	76.74 ± 13.21	33.57 ± 7.97	43.17 ± 8.13	68.89 ± 16.23	3.22 ± 2.48
University and above	22(3.3)	81.05 ± 19.20	36.86 ± 11.31	44.18 ± 9.74	75.64 ± 12.37	3.41 ± 2.28
**Marital status**						
Unmarried	131(19.4)	72.61 ± 14.08	31.42 ± 6.54	41.19 ± 9.45	61.28 ± 12.78	4.01 ± 2.86
Married	543(80.6)	75.03 ± 13.39	32.83 ± 7.31	42.20 ± 8.14	64.78 ± 12.87	3.18 ± 2.17
**Monthly family income (RMB)**						
< 5000	234(34.7)	73.22 ± 13.49	31.66 ± 7.09	41.56 ± 8.53	61.14 ± 11.86	3.32 ± 2.26
5000-10,000	281(41.7)	73.49 ± 13.23	32.46 ± 7.07	41.03 ± 8.14	63.54 ± 10.91	3.62 ± 2.54
> 10,000	159(23.6)	78.42 ± 13.53	34.04 ± 7.32	44.38 ± 8.31	69.43 ± 15.82	2.89 ± 2.04
**Self-rated health**						
Poor	77(11.4)	66.61 ± 12.02	30.10 ± 5.21	36.51 ± 8.87	58.44 ± 11.47	6.27 ± 3.35
Moderate	219(32.5)	70.89 ± 12.44	31.28 ± 6.84	39.61 ± 7.75	60.13 ± 10.43	3.75 ± 1.98
Good	378(56.1)	78.30 ± 13.20	33.79 ± 7.48	44.52 ± 7.74	67.55 ± 13.45	2.51 ± 1.65

Note: *RMB* Renminbi

### Correlations between social capital, health-promoting lifestyles, and frailty

Pearson’s *r* correlation analysis indicated that social capital as well as structural and cognitive social capital were all significantly positively associated with health-promoting lifestyles, and were significantly negatively associated with frailty and its three dimensions. Moreover, health-promoting lifestyles were also significantly negatively associated with frailty and its three dimensions (Table 2).

**Table 2:** Correlations (r) between social capital, health-promoting lifestyles, and frailty (*N* = 674)

Variables	Mean ± SD	1	2	3	4	5	6	7	8
1. Social capital	74.56 ± 13.55	1							
2. Structural social capital	32.55 ± 7.18	0.844***	1						
3. Cognitive social capital	42.01 ± 8.41	0.889***	0.506***	1					
4. Health-promoting lifestyles	64.10 ± 12.92	0.548***	0.434***	0.513***	1				
5. Frailty	3.34 ± 2.35	−0.333***	−0.196***	−0.370***	− 0.313***	1			
6. Physical frailty	1.62 ± 1.04	− 0.234***	−0.135***	− 0.262***	− 0.242***	0.864***	1		
7. Psychological frailty	1.14 ± 1.03	−0.285***	− 0.170***	− 0.314***	− 0.283***	0.700***	0.319***	1	
8. Social frailty	1.16 ± 0.55	−0.197***	−0.118**	− 0.216***	−0.090*	0.401***	0.135***	0.165***	1

Note: *p < 0.05, **p < 0.01, ***p < 0.001

### Linear regression analysis for the association between social capital and frailty

The results of the linear regression analysis indicated that the associations between structural social capital and frailty and its three dimensions was not statistically significant after adjusting for demographic factors. However, cognitive social capital was significantly negatively associated with frailty and its three dimensions. The details of all linear regression results are shown in Table 3.

**Table 3:** Linear regression analysis for the association between social capital and frailty (*N* = 674)

Variables	Frailty			Physical frailty			Psychological frailty			Social frailty		
	Beta	*t*	*P*	Beta	*t*	*P*	Beta	*t*	*P*	Beta	*t*	*P*
Age	0.057	1.779	0.076	0.113	3.382	0.001	−0.043	−1.186	0.236	−0.008	−0.220	0.826
Sex	0.015	0.474	0.635	0.063	1.849	0.065	−0.081	−2.187	0.029	0.033	0.864	0.388
Residence	−0.288	−7.833	< 0.001	−0.304	−7.959	< 0.001	− 0.151	−3.614	< 0.001	− 0.045	− 1.042	0.298
Education level	− 0.100	−2.762	0.006	− 0.098	−2.593	0.010	− 0.091	− 2.194	0.029	0.032	0.739	0.460
Marital status	−0.075	−2.357	0.019	−0.029	−0.893	0.372	< 0.001	0.005	0.996	−0.232	−6.179	< 0.001
Monthly family income	−0.030	−0.912	0.362	0.008	0.243	0.808	−0.062	−1.652	0.099	−0.036	−0.927	0.354
Self-rated health	−0.347	−10.059	< 0.001	−0.340	−9.452	< 0.001	− 0.208	−5.286	< 0.001	−0.089	−2.165	0.031
Structural social capital	0.017	0.464	0.643	0.019	0.501	0.616	0.004	0.107	0.915	0.008	0.177	0.859
Cognitive social capital	−0.226	−6.034	< 0.001	− 0.128	−3.269	0.001	− 0.220	− 5.153	< 0.001	−0.175	−3.942	< 0.001
*R*^*2*^	0.369			0.317			0.178			0.113		
*F*	43.056***			34.197***			16.026***			9.406***		

Note: ***p < 0.001, beta = standardized regression coefficients, *R*^*2*^ *=* coefficient of determination

### Mediation test for health-promoting lifestyles

**Fig. 2:**
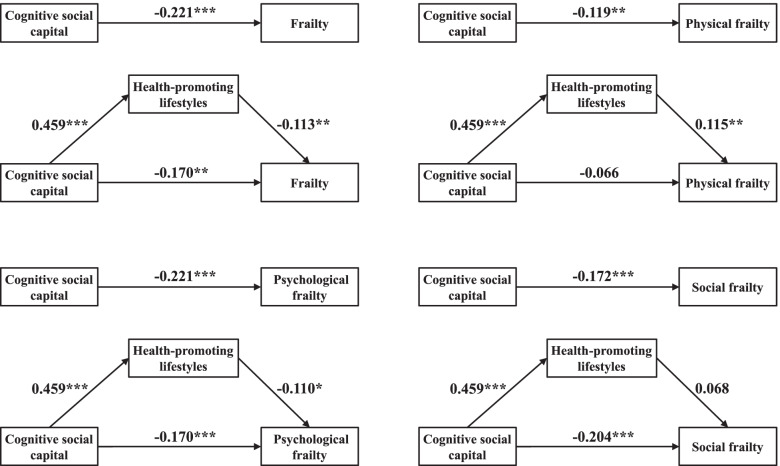
Health-promoting lifestyles mediated the association of cognitive social capital with frailty, physical frailty, psychological frailty, and social frailty. Note: **p < 0.01, ***p < 0.001

We constructed four models to test the mediating role of health-promoting lifestyles in the associations between cognitive social capital and frailty and its three dimensions (Fig. 2, Table 4). Health-promoting lifestyles played a mediating role in the associations between cognitive social capital and frailty, as well as the physical and psychological frailty dimensions, but not social frailty.

**Table 4:** Testing the Mediation effect of health-promoting lifestyles

Path	Effect	*Beta*	*SE*	*95%CI*	*P*
Cognitive social capital → health-promoting lifestyles → frailty	Total effect, c	−0.221	0.037	−0.292 ~ − 0.146	< 0.001
	Direct effect, c’	− 0.170	0.042	−0.252 ~ − 0.091	< 0.001
	Indirect effect, a*b	− 0.052	0.016	− 0.084 ~ − 0.019	0.002
	Ratio of indirect to total effect mediated (a*b/c)	23.5%			
Cognitive social capital → health-promoting lifestyles →physical frailty	Total effect, c	−0.119	0.039	−0.194 ~ − 0.040	0.005
	Direct effect, c’	− 0.066	0.044	− 0.152 ~ 0.019	0.123
	Indirect effect, a*b	−0.053	0.017	−0.086 ~ − 0.020	0.002
	Ratio of indirect to total effect mediated (a*b/c)	44.5%			
Cognitive social capital → health-promoting lifestyles → psychological frailty	Total effect, c	−0.221	0.037	−0.293 ~ − 0.150	< 0.001
	Direct effect, c’	− 0.170	0.042	− 0.257 ~ − 0.090	< 0.001
	Indirect effect, a*b	−0.051	0.019	−0.090 ~ − 0.012	0.010
	Ratio of indirect to total effect mediated (a*b/c)	23.1%			
Cognitive social capital → health-promoting lifestyles → social frailty	Total effect, c	−0.172	0.043	−0.251 ~ − 0.084	< 0.001
	Direct effect, c’	− 0.204	0.049	− 0.297 ~ − 0.107	< 0.001
	Indirect effect, a*b	0.031	0.020	− 0.005 ~ 0.072	0.096
	Ratio of indirect to total effect mediated (a*b/c)	NA			

Note: *beta* standardized coefficient, *CI* confidence interval, *SE* standardized error, *NA* not applicable

## Discussion

This study examined the relationship between social capital and frailty in older adults, and explored the mediating effect of health-promoting lifestyles in this relationship. The results showed that cognitive social capital was significantly negatively associated with frailty and its three dimensions (physical, psychological, and social frailty), but structural social capital was not. In addition, health-promoting lifestyles played a mediating role in the associations between cognitive social capital and frailty, as well as the physical and psychological frailty dimensions, but not social frailty.

Our results indicated that after controlling for demographic factors, cognitive social capital was significantly negatively associated with frailty and its three dimensions (physical, psychological, and social frailty). In other words, individuals with higher trust, cohesion, and reciprocity were less likely to suffer from frailty. This result partially confirmed Hypothesis 1, and the result was similar to that of a previous study [29]. Longitudinal studies in Japan have found that older adults who reverse the frailty process have a higher degree of trust in their neighbors and have closer relationships with them, which may assist in maintaining and promoting older adults’ physical, psychological and social functions [29]. In addition, a previous study also revealed that greater social cohesion can prevent frailty among older adults in the community [54]. Social cohesion is the result of a complex process involving factors such as the quality of the neighborhood, the individual’s mental and physical condition, the individual’s subjective assessment of the community, and the nature and quality of social contacts [55]. A more cohesive society may be more likely to organize various activities, such as health education and physical exercise, to provide residents with more opportunities to maintain and promote health [56]. This implies that individuals who perceive greater cohesion may be more willing to participate in such activities. In this process, physical fitness is strengthened and psychological and social needs are met, resulting in the possibility of frailty being reduced. Reciprocity refers to the ability to give something in return for receiving, and embodies mutual help and support between individuals [57]. A previous study found that recognizing the norms of reciprocity can prevent or reduce the loss of frail older adults’ own resources, thereby promoting their health [58]. In addition, reciprocity can encourage individuals to participate in voluntary services and sports activities [59], and promote the dissemination of health information [60], all of which are beneficial for reducing frailty in older adults.

However, inconsistent with the results of previous studies that indicated that social activities influenced frailty [29], the relationship between structural social capital and frailty was not statistically significant. A previous study has suggested that links between social structure and health largely depend on the quality of self-experience in the individual’s life [61]. In other words, if individuals with higher structural social capital do not have good cognition and experience of the social environment, they still cannot use those social resources to benefit their health. Previous studies have also suggested that structural social capital and cognitive social capital have different effects on health outcomes [62]. This is mainly reflected in the fact that structural social capital affects health through participation in social activities [63], while cognitive social capital does this through controlling risky behaviors, reducing stress and providing mutual assistance and support [64, 65]. In addition, previous studies also suggested that cognitive social capital is a stronger protective factor for depressive symptoms than structural social capital, and cognitive social capital mediates the relationship between structural social capital and depressive symptoms. It may be inferred from this that the relationship between structural social capital and frailty may be hidden by cognitive social capital. In view of the lack of relevant research evidence, further exploration is needed in this regard.

This study also found that health-promoting lifestyles mediated the associations of cognitive social capital with frailty, and the physical and psychological frailty dimensions. This study preliminarily elucidated the mechanism of association between cognitive social capital and frailty, and provided a scientific basis for further targeted intervention. This mediating effect was mainly divided into two stages, namely, 1) cognitive social capital had a positive impact on health-promoting lifestyles, and 2) health-promoting lifestyles had a negative impact on frailty. Both of these stages have been confirmed in previous studies [40, 66–68], which provides sufficient literature support for this study. Individuals with higher cognitive social capital have a stronger sense of social identity and closer neighborhood relationships, that enable them to encourage each other to cultivate a healthy lifestyle [69], and the improvement of lifestyle promotes physical and psychological health [70], resulting in a lower risk of frailty. It is worth noting that there was no statistically significant association between the health-promoting lifestyles and the social frailty dimension, that led to the failure of the mediation effect. This may have been because the measurement content of social frailty in this study was whether participants lived alone, their desire for others’ company and whether they received help from others. Logically speaking, whether individuals adopted a healthy lifestyle did not appear to affect their social frailty in this study. This result lacks literary support, and further exploration is necessary in the future.

This study has important scientific and practical significance for the prevention and intervention of frailty. First, this study supplements the literature elucidating the association between social capital and frailty in older adults. Second, to our best knowledge, this is the first study to explore the mediating of health-promoting lifestyles in the association between the social capital and frailty. It presents novel discovery on the mediating mechanism underpinning the association between the social capital and frailty among older adults. Third, this study can provide new ideas for the prevention and intervention of frailty. Findings of this study suggest that in the future, we can implement interventions on frailty from the perspective of cognitive social capital and health-promoting lifestyles. Chinese community health care workers can consider formulating community frailty intervention plans. On the one hand, they can enhance the cognitive social capital of the older adults by organizing collective health-related activities and building community mutual assistance platforms. On the other hand, they can strengthen health education and guide the older adults to develop healthy lifestyles such as physical exercise and reasonable diet, thereby reducing the occurrence of frailty.

This study has some limitations. First, our research is cross-sectional, which makes it impossible to clarify the causal relationship and the direction of effect among social capital, health-promoting lifestyles, and frailty. In the future, longitudinal studies or intervention studies can be conducted to further confirm the longitudinal association or causality between them. Second, the social capital assessment tool used in this study is different from previous research, so we need to be cautious about the comparison of study results with previous study results. In the future, the same assessment tools as this study can be used to further verify the results of this study. Third, all surveys were self-reported, and that may have introduced information bias. Finally, our sample was only selected from one city in China, so the research results may not be generalizable to older adults in other countries and regions.

## Conclusions

Higher cognitive social capital was associated with a reduced likelihood of frailty. The health-promoting lifestyles partially mediated the association between cognitive social capital and frailty. The use of health-promoting lifestyles or appropriate cognitive social capital interventions may reduce frailty among older adults.

## Supplementary Information

**Additional file 1.** Demographic information questionnaire.

## Data Availability

The datasets can be made available to any interested person(s) contacting the corresponding author via email.

## Abbreviations

CSSCS: Chinese Shortened Social Capital Scale
HPLP-S: Simplified version of the Health-Promoting Lifestyle Profile
TFI: Tilburg Frailty Indicator
SD: Standard Deviation
SEM: Structural Equation Models
CI: Confidence Interval
RMB: Renminbi
