# Supplementary material for: Association between social capital and frailty and the mediating effect of health-promoting lifestyles in Chinese older adults: a cross-sectional study
Source: BMC Geriatr. 2022 Mar 2;22:175. doi: 10.1186/s12877-022-02815-z (PMC8889641; doi:10.1186/s12877-022-02815-z)
Supplement: Supplementary file 1 — Additional file 1. Demographic information questionnaire. [file 12877_2022_2815_MOESM1_ESM.docx]

## Additional file 1 Demographic information questionnaire

**1. Your age (years):**

①60-69

②70-79

③≥80

**2.Sex:**

①Male

②Female

**3.Your residence:**

①Urban

②Rural

**4. Educational level:**

①primary school and below

②junior middle school

③high school

④university and above

**5.Marital status:**

①Unmarried

②Married

**6.** **What is your monthly family income?**

①<5,000

②5,000-10,000

③ >10,000

**7.How do you think your health status:**

①Poor

②Moderate

③Good
